# Reduced left dorsolateral prefrontal activation during spatial imagery in elite snowboard halfpipe athletes

**DOI:** 10.3389/fpsyg.2026.1817277

**Published:** 2026-04-23

**Authors:** Zhuorui Jiang, Jiacheng Chen, Yanzhang Chen, Chenglin Zhou, Wenzi Wang

**Affiliations:** 1 School of Business, Xianda College of Economics & Humanities Shanghai International Studies University, Shanghai, China; 2 College of Education for the Future, Beijing Normal University at Zhuhai, Zhuhai, China; 3 School of Psychology, Shanghai University of Sport, Shanghai, China

**Keywords:** inhibitory control, mental rotation, neural processing pattern, snowboard halfpipe, spatial imagery

## Abstract

**Objective:**

This study investigates whether elite snowboard halfpipe athletes exhibit different prefrontal activation patterns during spatial imagery processing, with particular attention to whether these patterns are compatible with neural efficiency and to the regulatory role of the dorsolateral prefrontal cortex (DLPFC) in the interaction between spatial perception and inhibitory control.

**Methods:**

Using a single-factor between-subjects design, 10 National Team snowboard halfpipe athletes in the expert group and 15 collegiate novice participants in the novice group were recruited. Functional near-infrared spectroscopy (fNIRS) was employed to monitor oxygenated hemoglobin (HbO) concentration changes in the prefrontal cortex while participants performed mental rotation-based spatial perception and spatial response inhibition tasks.

**Results:**

Behavioral data indicated no significant differences in reaction time or accuracy between groups, with task performance exhibiting a ceiling effect. However, neuroimaging results revealed that the expert group exhibited significantly lower HbO activation levels in the left DLPFC compared to the novice group while maintaining equivalent behavioral performance.

**Conclusion:**

Snowboard halfpipe athletes exhibited a different neural processing pattern during spatial imagery tasks, characterized by lower left-DLPFC HbO responses under comparable behavioral performance. This pattern is compatible with, but does not by itself prove, greater neural efficiency, and alternative explanations such as differences in task engagement, strategy use, and baseline cognitive ability should also be considered.

## 1 Introduction

Snowboard halfpipe is an extreme winter sport that integrates high speed, complex aerial maneuvers and precise landings. Athletes must execute sophisticated aerial skills across four phases—approach, take-off, flight, and landing—within a constrained spatial environment (Turnbull et al., 2011). During flight, athletes often perform multi-axis body rotations (e.g., off-axis twists and somersaults), placing exceptional demands on spatial orientation. To maintain balance and precisely regulate posture at high velocity, athletes must generate accurate spatial imagery, defined as the ability to process and manipulate

spatial information in working memory in the absence of external sensory input.

Mental rotation is a canonical paradigm for assessing spatial imagery ability and is widely used to quantify athletes' spatial cognition. Embodied cognition accounts suggest that motor expertise can substantially facilitate mental rotation performance (Voyer and Jansen, 2017). Prior work has shown that elite athletes in closed-skill sports such as gymnastics and diving—who undergo extensive aerial rotation training—typically outperform non-athletes in mental rotation tasks, exhibiting faster responses and/or higher accuracy (Schmidt et al., 2016). Compared with indoor sports, however, snowboard halfpipe additionally requires rapid adaptation to dynamic outdoor constraints (e.g., terrain and snow conditions), which may necessitate distinct neurocognitive strategies.

Evidence indicates that athletes with extensive multi-axis rotation experience tend to show better mental rotation performance and stronger activation in task-relevant brain regions, supporting a close link between rotational experience and spatial cognition (Feng et al., 2024a,b; Li and Smith, 2021). Training involving complex rotations can further enhance spatial cognition and mental rotation ability, which is critical for maintaining balance and controlling posture during high-speed aerial phases (Castañer et al., 2018). This link is especially relevant to snowboard halfpipe because performance quality depends heavily on the control of airtime, take-off, landing, and rotational amplitude; recent field analyses of elite halfpipe and snowboard freestyle performance likewise identify rotation amount and timing around take-off/landing as key performance parameters (Gorges et al., 2024, 2025). Unlike many laboratory mental rotation tasks, halfpipe athletes must continuously update body orientation, anticipate landing alignment, and adapt these internal transformations to changing environmental constraints such as wall geometry, snow condition, and speed. Such demands imply that halfpipe expertise relies not only on spatial imagery *per se*, but also on executive processes that maintain task goals, suppress inappropriate responses, and rapidly remap perception to action. Moreover, complex spatial tasks such as rotated-map processing recruit prefrontal and parietal areas, reflecting the neural control demands of spatial information processing (Ou et al., 2025). Collectively, these findings suggest that prolonged sport-specific training drives functional adaptations in perceptual–motor systems, potentially yielding more efficient neural processing in sport-relevant cognitive tasks (Yarrow et al., 2009).

The neural efficiency hypothesis provides a theoretical framework for such expertise-related advantages. It posits that experts often exhibit lower cortical activation than novices when performing domain-relevant tasks, reflecting more efficient use of neural resources (Li and Smith, 2021; Neubauer and Fink, 2009). Although visual imagery and visual perception share overlapping neural substrates across visual, parietal and prefrontal cortices, imagery lacks bottom-up sensory input and therefore depends more strongly on top-down control over internally maintained representations (Clément and Tallon-Baudry, 2025; Dijkstra et al., 2019; D'Esposito and Postle, 2015). In particular, the dorsolateral prefrontal cortex (DLPFC), a core hub within cognitive control networks, plays a central role in action planning, inhibitory control and spatial information processing by supporting the active maintenance and manipulation of spatial information in working memory, biasing attention toward task-relevant internal representations, and contributing to response selection when prepotent actions must be inhibited (Funahashi, 2017; Miller and Cohen, 2001). For snowboard halfpipe athletes, these functions should be repeatedly engaged when athletes mentally simulate body orientation during aerial rotation while simultaneously suppressing premature or incorrect responses and updating action plans for landing. Therefore, if elite halfpipe training fosters proceduralized and economical spatial processing, neural efficiency should be expressed as reduced DLPFC recruitment despite preserved behavioral performance.

Prior sport-neuroscience studies likewise document expertise-related differences in cortical activity, sensorimotor rhythm activity, neurocognitive preparation, and functional connectivity during visuomotor performance, including complex golf, dart-throwing, and shooting tasks (Baumeister et al., 2008; Cheng et al., 2015; Cooke et al., 2014; Haufler et al., 2000; Wang et al., 2020). Longitudinal and cross-sectional evidence further suggests that expertise-related refinement may involve progressive reorganization of functional brain connectivity across learning stages rather than a simple monotonic reduction in activation (Chen et al., 2022; Wang et al., 2024). More recent EEG evidence also indicates that intermediate performers can exhibit greater psychomotor efficiency than true novices during visuomotor control, underscoring that neural refinement may emerge gradually with training (Lu et al., 2025). It remains unclear, however, whether snowboard halfpipe athletes similarly conform to the neural efficiency hypothesis during spatial processing—especially under conditions that require inhibitory control—and what DLPFC activation patterns characterize their performance.

Functional near-infrared spectroscopy (fNIRS) is well suited to address this question because it is portable, relatively tolerant of movement constraints, and enables measurement of cortical hemodynamic changes in more naturalistic settings (Ferrari and Quaresima, 2012; Herold et al., 2018). Methodological reviews provide guidance for fNIRS acquisition, preprocessing and interpretation (Scholkmann et al., 2014), and spatial registration work based on the international 10-20 system and MNI coordinates supports cross-participant and cross-study localization of fNIRS channels (Okamoto et al., 2004; Tsuzuki et al., 2007).

Accordingly, this study used fNIRS to compare prefrontal hemodynamic responses between national-team snowboard halfpipe athletes in the expert group and non-athlete university students in the novice group during mental-rotation-related spatial perception and spatial response inhibition. We aimed to: (1) characterize behavioral features of spatial imagery in elite halfpipe athletes; (2) test the applicability of the neural efficiency hypothesis in extreme-sport experts; and (3) clarify the regulatory role of the DLPFC during the interaction between spatial imagery and inhibitory control. Specifically, we hypothesized that: (H1) the expert group would show comparable or superior behavioral performance to the novice group on the spatial judgment and spatial response inhibition tasks; (H2) the expert group would exhibit lower task-evoked prefrontal activation, especially in the DLPFC, while maintaining similar behavioral output, consistent with neural efficiency; and (H3) because halfpipe expertise requires the integration of spatial imagery with executive control, group differences would be most apparent in DLPFC responses during tasks that combine spatial transformation with response regulation. Our findings provide neuroimaging evidence for brain plasticity in winter-sport athletes and may inform talent identification and cognitive training.

## 2 Materials and methods

### 2.1 Participants

Eleven national-team snowboard halfpipe athletes were initially recruited for the expert group and 16 age-matched non-sport university students for the novice group. Due to unexpected issues during near-infrared data acquisition, one expert and one novice participant had missing fNIRS data and were excluded from analysis. The final sample comprised 10 experts (three men, seven women) and 15 novices (eight men, seven women). Participants were 16–26 years old (overall mean age = 20.52 ± 3.35 years). The two groups did not differ significantly in age, and all participants were right-handed. Expert participants had more than 6 years of halfpipe-specific training (mean training duration 9.33 ± 4.01 years), held a National First-Class Athlete qualification or higher, and were active members of the national team. Novice participants were university students with no formal sports training background or competitive athletic experience. All participants reported good health, no history of psychotropic medication use or alcohol abuse, and normal or corrected-to-normal vision. Given the rarity of national-team halfpipe athletes, the attainable sample was necessarily modest but comparable to prior fNIRS studies using expert-novice designs in sport settings (e.g., Park et al., 2020; Yu et al., 2025; Chen et al., 2020). The study was approved by the Ethics Committee of Shanghai University of Sport (No. 102772019RT011) and conducted in accordance with the Declaration of Helsinki. Written informed consent was obtained from all participants, who could withdraw at any time.

### 2.2 Design

A one-factor between-subjects design was adopted, with group (experts vs. novices) as the independent variable. The dependent variables were response time, accuracy, and task-evoked prefrontal oxygenated hemoglobin (HbO) beta estimates.

### 2.3 FNIRS instrumentation

**Figure 1:**
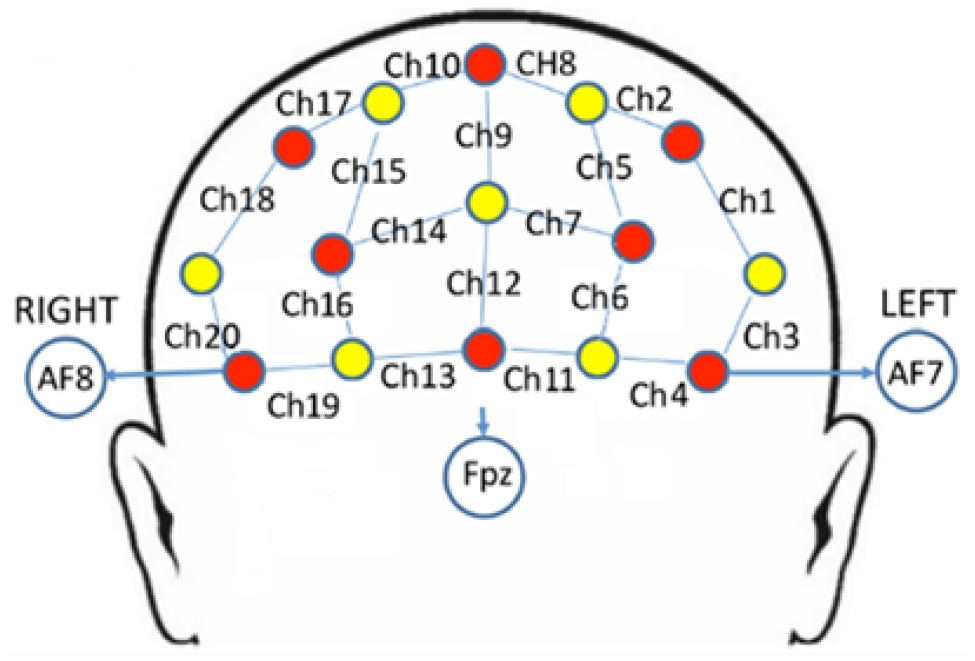
fNIRS channel layout over the prefrontal cortex. Yellow circles indicate detectors; red circles indicate sources. The central part of the probe array was positioned above Fpz, and the lowest row was aligned along AF7–Fp1–Fpz–Fp2–AF8 according to the international 10-20 system. Source-detector separation was 3 cm, and the midpoint of each source-detector pair was defined as the channel position. Lines connecting a source and a detector denote channels; numbers indicate channel indices.

A portable fNIRS system (NIRx Medizintechnik GmbH, Gustav-Meyer-Allee 25, Berlin, Germany) was used. An 8 × 7 montage (eight sources, seven detectors) operating at 780 and 830 nm yielded 20 channels over the prefrontal cortex. The probe set comprised eight emitters (red) and seven detectors (yellow) positioned over the frontal scalp. The center of the montage was placed above Fpz, and the lowest row of optodes was aligned along AF7–Fp1–Fpz–Fp2–AF8 in accordance with the international 10-20 EEG system (Figure 1). The source-detector separation was fixed at 3 cm, and the midpoint of each source-detector pair was defined as the corresponding channel location (Ch1–Ch20). This montage did not include short-separation channels. A compression cap was used to optimize scalp contact, and channel gain was maintained below seven to ensure signal quality. The sampling rate was 11.6258 Hz.

### 2.4 Tasks

Stimuli were presented using E-Prime 3.0. Each trial began with a central black fixation cross displayed for 700 ms, followed by a centrally presented stimulus for 1,000 ms and a 2,000 ms response window. The stimuli were cartoon images of a snowboard halfpipe athlete shown at four orientations (0°, 60°, 120°, and 180°) and facing either left or right. We used a sport-specific human figure rather than abstract objects because body-related stimuli map more closely onto the perspective-transformation demands relevant to aerial skill execution and are more likely to elicit expertise-related embodied processing (Feng and Li, 2021; Turnbull et al., 2011). The four angles were selected to sample low-to-high angular disparity within a standard mental-rotation range while keeping the number of trials manageable for fNIRS recording (Feng and Li, 2021). Two experimental tasks were administered: a spatial orientation judgment task and a spatial response inhibition task.

Spatial orientation judgment task: four blocks of 20 trials each. Participants imagined themselves as the cartoon athlete and judged the facing direction. If the athlete faced left, participants pressed “J” with the right index finger; if the athlete faced right, they pressed “F” with the right middle finger.

Spatial response inhibition task: two conditions (left-inhibition and right-inhibition) were administered. For example, in the left-inhibition condition, participants responded only to right-facing stimuli; left-facing stimuli required withholding a response, while keeping fingers resting on the relevant keys. Each condition comprised four blocks of 20 trials. Condition order was counterbalanced across participants.

### 2.5 Procedure

Participants first completed practice trials to ensure task comprehension. After practice, they completed the spatial orientation judgment task followed by the spatial response inhibition task while fNIRS data were recorded continuously. Adequate seated rest was provided between tasks, and the next task began only after the hemodynamic response had returned to baseline. During recording, participants were instructed to minimize head and body movement, avoid chewing and swallowing, and blink naturally.

### 2.6 Data processing

#### 2.6.1 Behavioral data processing

For both tasks, response time (correct trials only) and accuracy were extracted. To reduce the influence of outliers, trials with response times < 150 ms or >1,500 ms, as well as trials falling outside the mean ± 3 SD, were excluded from subsequent analyses.

#### 2.6.2 FNIRS preprocessing

Continuous fNIRS signals were recorded during task performance and preprocessed in MATLAB (MathWorks, Natick, MA, USA) using the Homer toolbox (MGH–Martinos Center for Biomedical Imaging, Boston, MA, USA). Processing steps were: (1) conversion of raw light intensity to optical density; subsequently, motion artifacts, including head movements, were identified and corrected. The hmrMotionArtifactByChannel function was employed to calculate the mean and standard deviation of the data within 1.5-s windows. If signal variations exceeded five times the standard deviation or 50 times the amplitude, the data within a 2-s window surrounding the event were flagged as motion artifacts and corrected using the hmrMotionCorrectSpline algorithm. Following this initial correction, a secondary artifact detection was performed applying the identical criteria; any trials containing residual, uncorrected artifacts were excluded from further analysis; (2) band-pass filtering to remove physiological noise and drift (0.01–0.10 Hz); and (3) concentration changes computed via the modified Beer–Lambert law (Cope et al., 1988), yielding HbO, HbR, and total hemoglobin. For inferential analyses, we focused on HbO. This choice was based on methodological and physiological considerations: HbO generally shows larger task-evoked amplitude changes and higher sensitivity to regional cerebral blood-flow increases than HbR, often yielding a higher signal-to-noise ratio in cognitive fNIRS studies (Hoshi, 2007; Pinti et al., 2020). In addition, across simultaneous fNIRS–fMRI comparisons, HbO has shown the strongest correspondence with BOLD responses among the optical indices (Cui et al., 2011; Strangman et al., 2002). We therefore used HbO as the primary activation metric, consistent with common reporting practice in the fNIRS literature (Kinder et al., 2022; McCraw et al., 2022).

For each condition, HbO values were averaged across the peak window of the hemodynamic response (2–9 s after task onset) for each channel. Channels were assigned to cortical regions using virtual spatial registration based on the international 10-20 system and source-detector midpoint locations, following the 10-20/MNI registration approach of Okamoto et al. (2004) and Tsuzuki et al. (2007). Anatomical labels therefore reflect standard-space estimates rather than participant-specific 3D digitization. Six regions of interest (ROIs) were defined, and HbO values were averaged across channels within each ROI (Table 1).

**Table 1:** Mapping between fNIRS channels and prefrontal ROIs.

Prefrontal ROI	Hemisphere	Channels
Ventrolateral prefrontal cortex (VLPFC)	Left	1, 3, 4
Ventrolateral prefrontal cortex (VLPFC)	Right	18, 19, 20
Dorsolateral prefrontal cortex (DLPFC)	Left	2, 5, 7, 8, 9
Dorsolateral prefrontal cortex (DLPFC)	Right	10, 14, 15, 17
Frontopolar prefrontal cortex (FPA)	—	6, 12, 16
Orbitofrontal cortex (OFC)	—	11, 13

### 2.7 Data analysis

Behavioral analyses were conducted in SPSS 26.0 (IBM SPSS Statistics, IBM Corp., Armonk, NY, United States) using independent-samples *t*-tests to compare the expert and novice groups on response time and accuracy for each task condition. For ROI-based HbO data, 2 (group: expert, novice) × 3 (condition: orientation judgment, right inhibition, left inhibition) mixed ANOVAs were performed separately for each ROI. For each effect of interest (group, condition, and group × condition interaction), ROI-wise *p* values were adjusted for multiple comparisons across the six ROIs using the Benjamini–Hochberg FDR procedure (*q* < 0.05). When significant interactions were observed, simple-effects analyses were planned. Before parametric testing, normality and homogeneity of variance were examined and met. Behavioral data are reported as mean ± standard error with Cohen's *d*, whereas fNIRS results are reported as mean ± standard deviation with *F*-values and partial eta squared ($\eta_{p}^{2}$).

## 3 Results

### 3.1 Behavioral performance

In the spatial orientation judgment task, the expert group showed slightly faster response times and slightly higher accuracy than the novice group; however, independent-samples *t*-tests indicated no significant between-group differences in response time or accuracy, and neither difference was significant and both effect sizes were trivial. In the spatial response inhibition task, the expert group showed slightly longer response times than the novice group in both inhibition conditions, whereas accuracy was marginally higher in athletes in the right-inhibition condition. None of these between-group differences reached statistical significance. Given the uniformly high accuracies, these behavioral null findings should be interpreted cautiously because ceiling effects and limited statistical power may have obscured small-to-moderate group differences. Descriptive statistics are presented in Table 2.

**Table 2:** Behavioral performance in the spatial orientation judgment and spatial response inhibition tasks.

Measure	Experts	Novices	*t*	*p*	95% CI	Cohen's *d*
Orientation judgment RT (ms)	453.27 ± 42.65	461.646 ± 71.173	−0.333	0.742	[−60.40, 43.65]	−0.136
Orientation judgment accuracy	93.90% ± 3.78%	92.53% ± 17.5%	0.242	0.811	[−10.33, 13.07]	0.099
Right-inhibition RT (ms)	223.71 ± 20.55	236.831 ± 33.399	−1.106	0.280	[−37.66, 11.42]	−0.452
Right-inhibition accuracy	98.00% ± 1.82%	97.80% ± 5.05%	0.120	0.906	[−3.26, 3.66]	0.049
Left-inhibition RT (ms)	225.05 ± 24.27	234.941 ± 31.682	−0.836	0.412	[−34.39, 14.61]	−0.341
Left-inhibition accuracy	96.80% ± 2.78%	97.87% ± 3.62%	−0.787	0.439	[−3.87, 1.73]	−0.323

### 3.2 FNIRS findings

**Figure 2:**
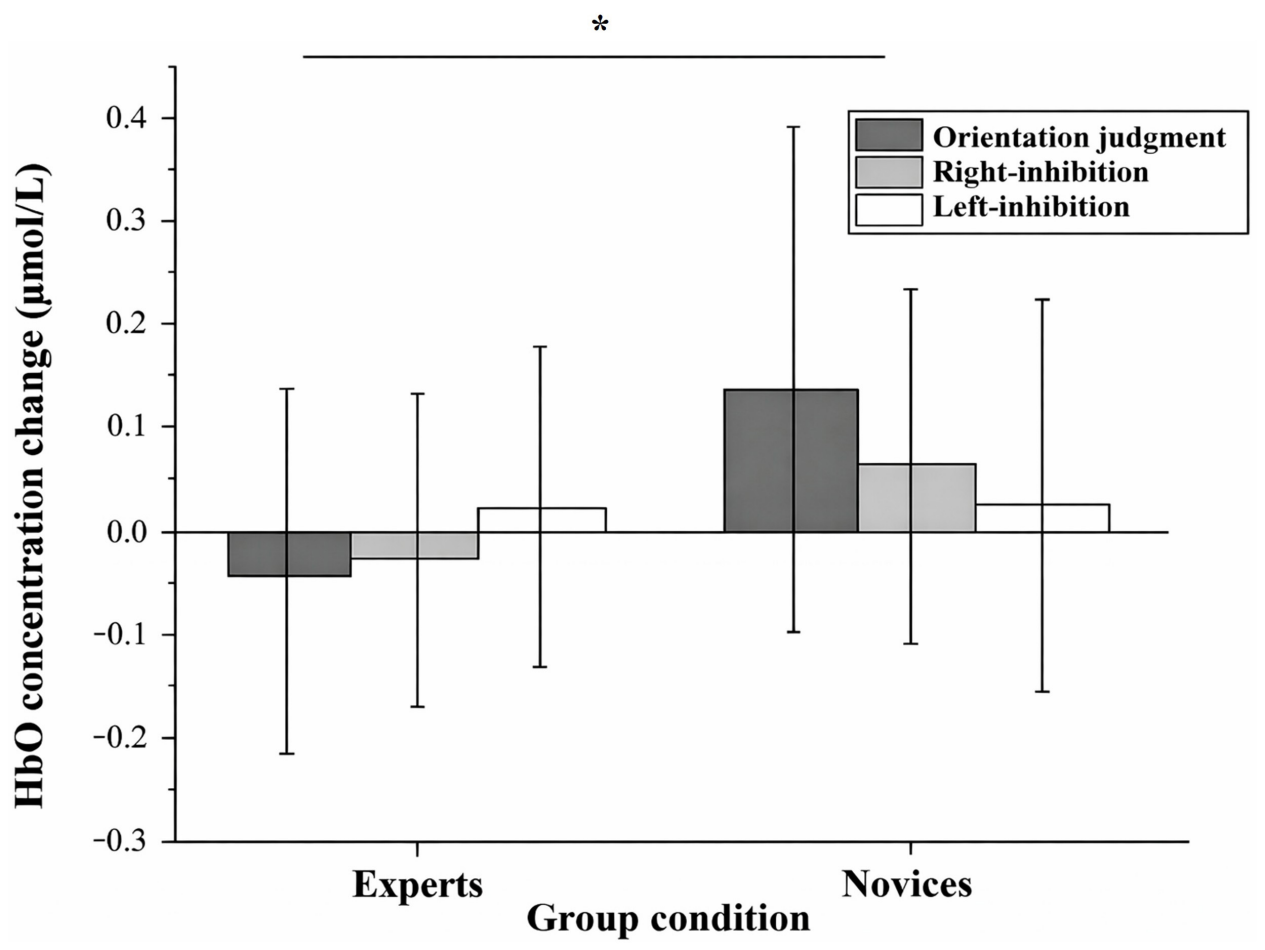
Task-evoked HbO concentration changes in the left DLPFC. Bars indicate mean values and error bars represent standard deviations. The significance markers in the figure denote a significant main effect of group, indicating that experts exhibited significantly lower left DLPFC HbO activation than novices *P < 0.50.

The HbO concentration data from the six ROIs during the experimental tasks were analyzed using a 2 (group: experts vs. novices) × 3 (condition: orientation judgment, right inhibition, left inhibition) mixed ANOVA. The results revealed a significant main effect of group, *F*_(1, 23)_ = 4.798, FDR-corrected *p* < 0.05, $\eta_{p}^{2}$ = 0.173. Specifically, as shown in Figure 2, the experts showed significantly lower HbO activation in the left DLPFC (−0.011 ± 0.033 μmol/L) than the novices (0.082 ± 0.027 μmol/L), with a 95% confidence interval for the mean difference of [−0.181, −0.005]. In contrast, the main effect of condition was not significant, *F*_(2, 22)_ = 0.128, *FDR*-corrected *p* > 0.05, $\eta_{p}^{2}$ = 0.011, nor was the interaction between group and condition, *F*_(2, 22)_ = 0.964, *FDR*-corrected *p* > 0.05, $\eta_{p}^{2}$ = 0.081. In addition, no significant or marginally significant main effects or interaction effects were observed in the other five ROIs.

## 4 Discussion

This study examined national-team snowboard halfpipe athletes using mental-rotation-related spatial orientation judgment and spatial response inhibition paradigms while recording prefrontal hemodynamics with fNIRS. The combination of comparable behavioral performance and lower left-DLPFC HbO responses suggests that expertise in this extreme-sport population is associated with an altered prefrontal processing profile even when overt performance differences are small. Consistent with recent work on axial-rotation expertise and rotated spatial information processing (Feng et al., 2024a; Ou et al., 2025), the present findings are best interpreted as preliminary evidence for a different neural processing pattern during laboratory spatial-imagery tasks, rather than definitive evidence of superior spatial imagery ability.

More specifically, the study contributes in three ways. First, it extends discussion of neural efficiency to an underexamined extreme-sport population. Second, by combining a sport-relevant mental-rotation paradigm with spatial response inhibition, it identifies the DLPFC as a plausible locus at which spatial transformation and executive control may interact in halfpipe expertise. Third, it shows that fNIRS can be used to study expertise-related neural adaptation in winter-sport athletes under relatively portable and sport-relevant conditions, providing a basis for more ecologically valid and longitudinal work.

### 4.1 Ceiling effects in behavioral performance

Both groups achieved high accuracies across the spatial judgment and inhibition tasks, and athletes tended to respond slightly faster overall, consistent with the embodied-cognition view that extensive rotational training can facilitate spatial transformation (Feng et al., 2024a,b; Voyer and Jansen, 2017). However, the absence of significant behavioral differences should not be interpreted as evidence of equivalence. With only 25 participants, the present design was primarily sensitive to large effects, so the null behavioral results may reflect limited power and Type II error. In addition, the tasks preserved some sport-relevant cues but remained static, two-dimensional, and rule-based; they did not reproduce the continuous three-dimensional posture updating, velocity information, risk constraints, and landing anticipation involved in real halfpipe performance (Turnbull et al., 2011). Together with the lack of pilot-based difficulty calibration, these features likely contributed to ceiling effects and reduced sensitivity to expertise-related behavioral differences.

Under such conditions, expertise-related variation may be more detectable at the neural than the behavioral level. Recent work indicates that orienteers processing rotated terrain information rely on specialized visuocognitive coupling strategies rather than simply faster reaction times (Ou et al., 2025), and expert advantages are often clearest in tasks that closely match sport-specific demands (Yarrow et al., 2009). Likewise, multi-domain assessments suggest that expertise effects can be attenuated when tasks lack specificity or situational pressure (Vona et al., 2024). Thus, the present pattern of comparable performance with lower prefrontal activation is compatible with a neural-efficiency account, but the ceiling-like behavioral results prevent strong inferences about superior spatial imagery ability in this task setting. Future studies should therefore adopt more sensitive and ecologically valid paradigms.

### 4.2 Interpreting reduced left DLPFC activation

The reduced HbO response in the left DLPFC in athletes is the central neurophysiological result. In the broader sport-neuroscience literature, lower task-related activation in experts is often discussed in relation to neural efficiency, but its interpretation depends on task familiarity, complexity, and the cognitive operations involved (Li and Smith, 2021; Yarrow et al., 2009). The DLPFC supports spatial working-memory manipulation, attention allocation, rule maintenance, response selection, and top-down control under inhibitory demands (Koechlin et al., 2003; Miller and Cohen, 2001). It also interacts with visuospatial and navigation-related systems (Diwadkar et al., 2000; Hagler and Sereno, 2006; Patai and Spiers, 2021). Accordingly, lower left-DLPFC responses in athletes suggest altered recruitment of domain-general executive control during these tasks. This interpretation converges with prior evidence that skilled performers often show more economical cortical dynamics during visuomotor preparation and execution, including reduced cortical activity in golf putting, expertise-related SMR modulation in dart throwing, and distinct EEG profiles in expert marksmen relative to novices (Baumeister et al., 2008; Cheng et al., 2015; Haufler et al., 2000). This may reflect more economical processing, but it may also be shaped by differences in task engagement, strategy use, or baseline cognitive ability.

Within the neural efficiency literature, lower cortical activation in experts has often been interpreted as reflecting more automated processing, more compact encoding, higher signal-to-noise ratios, and optimized resource allocation shaped by training (Li and Smith, 2021; Neubauer and Fink, 2009). At the same time, neural efficiency is known to depend on factors such as task difficulty, learning history, and the extent to which participants have developed efficient strategies (Neubauer and Fink, 2009). Halfpipe training repeatedly requires rapid online spatial simulation—continuous updating of body orientation, trajectory prediction, and landing estimation within narrow time windows. Such demands may foster stable transformation schemas, shifting mental rotation from effortful prefrontal computation toward faster, more perceptualized processing. This interpretation is also compatible with evidence for psychomotor refinement and learning-related reorganization of functional connectivity across skill levels in golfers and other visuomotor performers (Chen et al., 2022; Lu et al., 2025; Wang et al., 2020, 2024). Because our fNIRS montage primarily covered the prefrontal cortex, we could not directly test parietal or visual contributions; nonetheless, the coexistence of preserved behavioral performance with reduced prefrontal recruitment is consistent with a neural adaptation pathway in which task-relevant networks outside the prefrontal cortex take on a greater share of processing (Dijkstra et al., 2019).

The group difference was primarily left-lateralized. Because the left DLPFC is often implicated in rule maintenance, serial operations, and response selection, reduced activation may indicate more automatized stimulus–response mapping and reduced explicit control in athletes. However, other explanations remain plausible, including group differences in engagement, preferred problem-solving strategies, or other unmeasured individual characteristics. Prior work on skilled motor learning also reports lateralized modulation of DLPFC–motor interactions (Cao et al., 2020), which is broadly compatible with the present pattern.

Importantly, neural efficiency should not be reduced to the idea that lower activation is always better. Rather, efficient recruitment depends on the match between available resources and task demands; under novelty, uncertainty, or pressure, experts may show greater or more distributed activation to support monitoring and strategic control (Carius et al., 2023). In the present rule-based and relatively easy tasks, a low-activation pattern may reflect automatization. Descriptively, group separation appeared somewhat larger in the spatial judgment condition than in the inhibition condition, although the interaction was not significant, suggesting that halfpipe expertise may map more directly onto spatial transformation than inhibition *per se*.

These findings have only preliminary practical implications. For training, combining sport-specific imagery practice (e.g., action imagery, trajectory prediction, and reference-frame transformations) with progressively increasing control demands may promote automatization and robust efficiency under load. Additionally, as wearable fNIRS advances, prefrontal HbO-based neurofeedback shows promising potential. However, both approaches require further validation through rigorous randomized controlled trials and longitudinal studies (Klein et al., 2024) before firm applied recommendations can be made.

### 4.3 Limitations and future direction

This study has several limitations. First, sample size was necessarily modest because national-team halfpipe athletes are rare; however, this small sample substantially limits statistical power, precision, and generalizability. In particular, the study was primarily powered to detect large between-group effects, so the null behavioral findings should not be interpreted as evidence of true equivalence and may instead reflect Type II error. Second, the behavioral tasks were relatively simple and ceiling-prone. Although they retained some sport-relevant cues, they did not capture the dynamic, three-dimensional, and risk-laden demands of real halfpipe performance, thereby weakening the interpretation of neural differences in the absence of behavioral separation. Third, the fNIRS montage was restricted to the prefrontal cortex and did not include short-separation channels. Consequently, we could not examine parietal, cerebellar, or sensorimotor contributions, test network-level mechanisms, or fully separate cortical signals from extracerebral/systemic components. Fourth, although the novice group consisted of university students without formal sports training, we did not systematically measure or match groups on education level, general cognitive ability, habitual physical activity, or anthropometric characteristics. Finally, the age range (16–26 years) spanned late adolescence to early adulthood, a developmental window during which prefrontal circuitry and executive functions continue to mature (Kolk and Rakic, 2022; Tervo-Clemmens et al., 2023). This developmental heterogeneity may have introduced additional variability in cortical activation patterns.

Future work should prioritize: (1) longitudinal or quasi-experimental designs to track changes in neural processing across training stages and relate them more directly to sport performance; (2) more sensitive and ecologically valid paradigms, such as VR- or video-based trajectory prediction, multi-rotation sequence planning, and tasks with calibrated difficulty, to reduce ceiling effects and test neural efficiency under realistic cognitive load, risk, and pressure; (3) broader cortical coverage, inclusion of short-channel regression, and multimodal approaches (e.g., EEG–fNIRS) to better characterize network dynamics and improve physiological specificity; (4) larger multi-center samples with systematic assessment of education, general cognitive ability, habitual physical activity, and anthropometric characteristics; and (5) narrower age ranges or statistical control of age-related neurodevelopmental differences.

## 5 Conclusions

Taken together, the present findings indicate that elite snowboard halfpipe athletes display a different prefrontal activation pattern during spatial imagery and response inhibition tasks, characterized by lower left-DLPFC HbO responses under comparable behavioral performance. This pattern is compatible with, but does not establish, neural efficiency. Given the small sample, ceiling-prone behavioral tasks, absence of short-channel regression, and limited cortical coverage, the results should be interpreted conservatively as preliminary evidence of a different neural processing pattern associated with expertise, rather than unequivocal evidence of superior spatial imagery ability.

## Data Availability

The original contributions presented in the study are included in the article/supplementary material, further inquiries can be directed to the corresponding author.
